# Genetic network and gene set enrichment analyses identify MND1 as potential diagnostic and therapeutic target gene for lung adenocarcinoma

**DOI:** 10.1038/s41598-021-88948-4

**Published:** 2021-05-03

**Authors:** Jinying Wei, Guangping Meng, Jing Wu, Qiang Zhang, Jie Zhang

**Affiliations:** 1 Department of Respiratory Medicine, The Second Affiliated Hospital of Jilin University, Changchun, 130041 China; 2 Department of General Practice, The First Hospital of Jilin University, Changchun, 130021 China

**Keywords:** Cancer, Cancer genetics, Cancer therapy

## Abstract

This study aimed to characterize the key survival-specific genes for lung adenocarcinoma (LUAD) using machine-based learning approaches. Gene expression profiles were download from gene expression omnibus to analyze differentially expressed genes (DEGs) in LUAD tissues versus healthy lung tissue and to construct protein–protein interaction (PPI) networks. Using high-dimensional datasets of cancer specimens from clinical patients in the cancer genome atlas, gene set enrichment analysis was employed to assess the independent effect of meiotic nuclear divisions 1 (MND1) expression on survival status, and univariate and multivariate Cox regression analyses were applied to determine the associations of clinic-pathologic characteristics and MND1 expression with overall survival (OS). A set of 495 DEGs (145 upregulated and 350 downregulated) was detected, including 63 hub genes with ≥ 10 nodes in the PPI network. Among them, MND1 was participated in several important pathways by connecting with other genes via 17 nodes in lung cancer, and more frequently expressed in LUAD patients with advancing stage (OR = 1.68 for stage III vs. stage I). Univariate and multivariate Cox analyses demonstrated that the expression level of MND1 was significantly and negatively correlated with OS. Therefore, MND1 is a promising diagnostic and therapeutic target for LUAD.

## Introduction

Lung cancer is the most frequent malignancy and responsible for the highest incidence and the largest number of deaths globally (approximately 1.8 million new cases and over 1 million deaths yearly)^1,2^. In China from 2008 to 2012 lung cancer was the leading cause of cancer-related death^3^, with the crude rates of incidence and deaths of 54.66/100,000 and 45.60/100,000, respectively. Squamous cell carcinoma and small cell lung cancer have been the most prevailing lung malignancy subtypes in the past; however, lung adenocarcinoma (LUAD) has recently emerged as the most common and most aggressive histological type^4^, with most cases being diagnosed at advanced stages. LUAD generally grows in the outer regions of the lungs for a long time before the appearance of symptoms, including mild insufficiency of breath, subtle weight loss, and a general sense of being unwell. A combination of imaging studies, including computed tomography (CT), magnetic resonance imaging (MRI), and positron emission tomography (PET), has been used to analyze lung cancer, whereas lung biopsy is generally required to diagnose the type of lung cancer. Early diagnosis and accurate staging for lung cancer are very important in planning an effective treatment regimen, particularly for the most aggressive cases of LUAD.

Gene-targeting therapeutic strategies are now emerging as potential treatments for LUAD. Targeted therapies and immunotherapy have been demonstrated to improve median survival times for a set of solid carcinomas in clinical trials^5^, resulting in long-term survival even for subjects with the advanced-stage lung carcinoma. However, progress related to the overall prognosis of LUAD has been limited, as the occurrence and development of this heterogeneous disease are regulated by different genes. The discovery of novel genes associated with the occurrence and progression of LUAD as well as effective diagnostic biomarkers is essential to characterizing the mechanisms underlying LUAD, identifying effective diagnostic biomarkers, and achieving significant breakthroughs for the precise diagnosis and effective treatment of LUAD.

Machine-based-learning approaches^4,6–12^ have been used to analyze both histological and molecular features of tumors for classification according to molecular patterns^9,12^ and identification of biologically relevant, tumor-type-defining and clinically informative genetic modifications^11^ in a variety of cancers. Nowadays, rapid development of “-omic” technologies, including gene chip analysis and next-generation sequencing, has been proven to generate vast volumes of molecular data for a tumor and publicly stored in databases for assessment of differentially expressed genes (DEGs) in a tumor without the need for such subjective diagnostics^13^. The Gene Expression Omnibus (GEO) Database was the first public database for high-throughput gene expression data as well as hybridization array, chip, microarray^14^, supporting MIAME-compliant data submissions. Another public database is the Cancer Genome Atlas (TCGA)^15^, which collects molecular data sets from exome sequencing, comparative genomic hybridization (CGH) arrays, DNA methylation arrays, RNA sequencing, and reverse protein phase arrays (RPPA) along with clinical information for cancers including LUAD. Thus, TCGA is a fundamental tool for the categorization and further study of the molecular pathogenesis for LUAD. Both the authoritative TCGA and GEO databases are publicly accessible through multiple platforms. Advances in computational tools have facilitated the use of machine-learning processes for analyzing histological data and molecular features, integrating both molecular analysis and visual inspection to enhance diagnostic power^9–12^. Therefore, machine-based learning approaches are the key development for switching from the original clinical diagnosis methods to a computer-based clinical diagnosis and categorization of tumors, and for optimizing treatment schemes to be ever-more personalized through characterization of an individual’s tumor.

In the present study, we downloaded genomic data from the GEO database to detect significant DEGs in LUAD and further validated these DEGs with transcriptomic data and clinicopathological data from TCGA database to investigate correlations between the expression of DEGs, including meiotic nuclear division 1 (MND1), and survival. The information retrieved for the DEGs was applied to construct a protein–protein interaction (PPI) network and conduct an overall survival (OS) analysis. In total, 495 DEGs were identified, of which MND1 was significantly associated with OS and thus might be used as a prognostic biomarker and a molecular curative target for LUAD. The results of the present study provide valuable information for understanding the mechanisms underlying the pathogenesis of LUAD and for the identification of diagnostic and therapeutic targets for LUAD.

## Results

### Clinicopathological statistics of TCGA LUAD cases

The clinic-pathological information for a total of 486 clinical LUAD samples downloaded from TCGA are listed in Table 1. The available clinic-pathological features for each LAUD case included the patient’s age at diagnosis (years), gender, carcinoma stage, and TNM grouping. Overall, 46% of the 486 LUAD cases were male and 54% were female, and their ages ranged from 33 to 88 years (Table 1). Notably, more than half of the LUAD cases (54.8%) were stage I at diagnosis, and only 5.2% were diagnosed at stage IV.

**Table 1:** Clinic-pathological features of LUAD patients in TCGA.

Clinical characteristics	Total (N = 486)	%
**Duration (d)**	769.6 (0–6812)	
**Age at diagnosis (y)**	66 (33–88)	
**Gender**		
Male	222	45.7
Female	264	
**Stage**		
I	262	54.8
II	112	23.5
III	79	16.5
IV	25	5.2
**T**		
T1	163	33.8
T2	260	53.8
T3	41	8.5
T4	19	3.9
**M**		
M0	333	93.3
M1	24	6.7
**N**		
N0	312	65.8
N1	90	19.0
N2	70	14.8
N3	2	0.4

### Identification of DEGs

A set of 495 DEGs was detected from three consolidated and batch-corrected datasets: GSE118370 (normal 6, tumor 6), GSE19188 (normal 15, tumor 18), and GSE40791 (normal 100, tumor 94), using |logFC|> 2 and *p* < 0.05 as the cutoff criteria. Among them, 145 DEGs were upregulated and 350 DEGs were downregulated.

**Figure 1:**
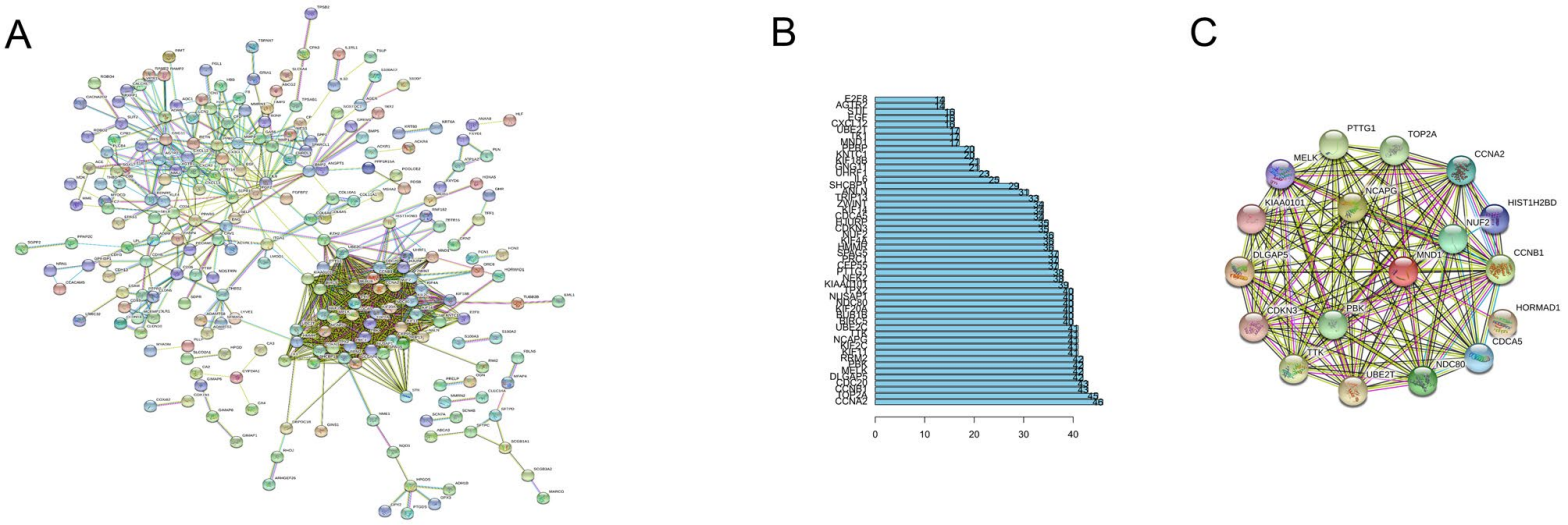
PPI networks of DEGs. (**A**) PPI network of DEGs constructed using STRING. (**B**) Top 50 hub genes. (**C**) MND1-connected PPI.

The co-expression, genetic, and physical interactions among the 495 DEGs and predicted genes were characterized using STRING to construct the PPI networks. A group of 63 hub genes had a node degree ≥ 10 in the PPI networks of upregulated and downregulated DEGs (Fig. 1A), suggesting that these genes play important roles in the progression of LUAD. The top 10 nodes in the PPI network were CCNA2, TOP2A, CCNB1, CDC20, DLGAP5, MELK, PBK, RRM2, K1F11, and K1F2C. However, for most of these hub genes, either no clinicopathological features were available in TCGA database or the relationship with clinical data was nonsignificant. Meaningful clinical data were available for MND1, which has 17 nodes (Fig. 1B) connected with other genes and participates in several pathways related to lung cancer (Fig. 1C), suggesting that MND1 might be essential in the progression of LUAD. Therefore, MND1 was elected as the target gene for further analysis in the present study.

### Expression of the MND1 gene

**Figure 2:**
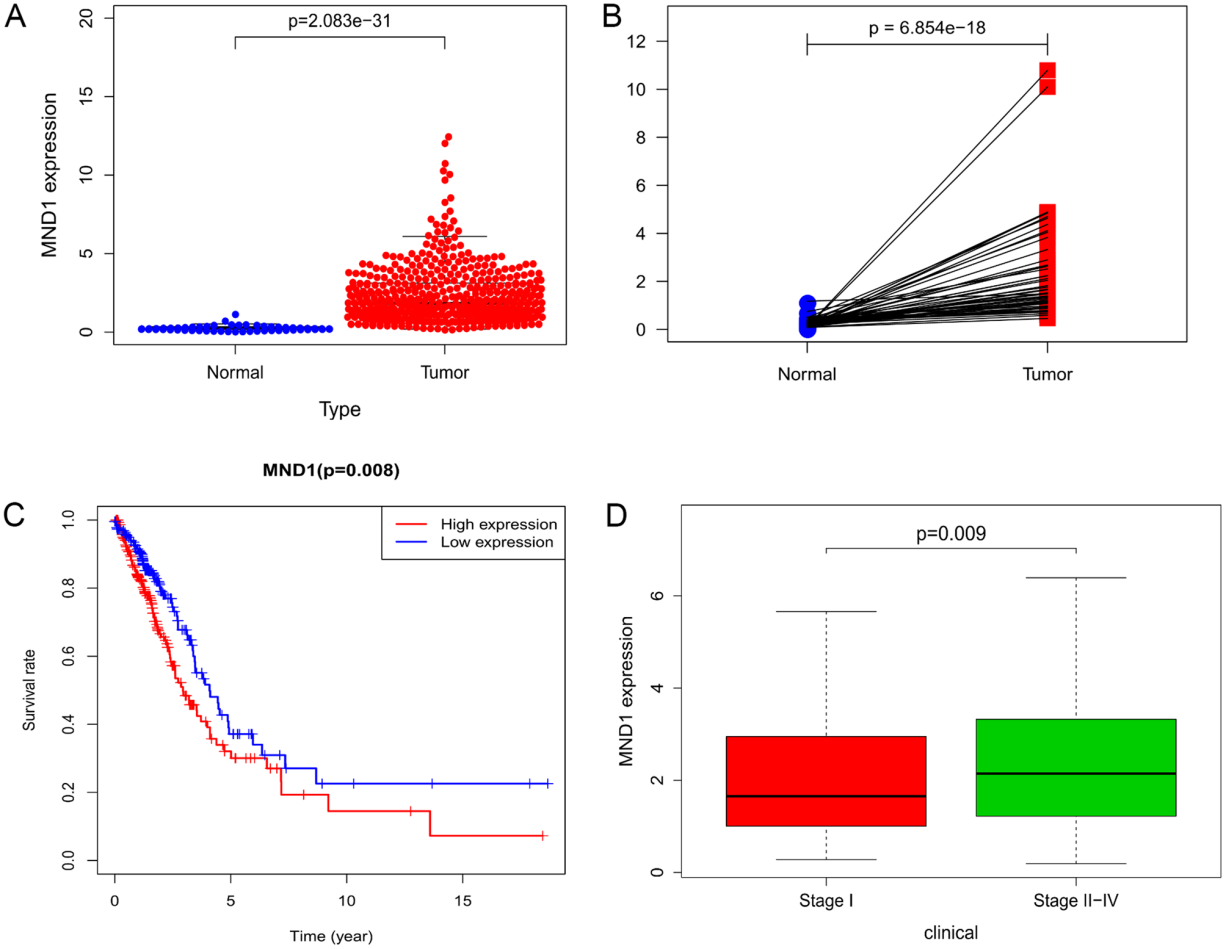
Associations of MND1 expression with clinic-pathological characteristics. (**A**) MND1 expression in healthy and LUAD tissues. (**B**) Different expression of MND1 in normal–LUAD paired tissues. (**C**) Survival rates with elevated and low expression of MND1. (**D**) Differential expression of MND1 between stage I and stage II–IV LUAD cases.

The differential scatter plot and paired difference analyses of MND1 expression showed a significant difference between 54 normal samples and 497 LUAD samples from TCGA database (Fig. 2A,B, *p* < 0.001). Compared with its expression in normal samples, MND1 was significantly upregulated in LUAD samples.

### MND1 expression and clinicopathological features

The associations between MND1 expression level and clinic-pathological data from TCGA were determined by logistic regression analysis (Table 2). When entered as a categorical dependent variable according to a median expression value of 1.83, MND1 expression was negatively related to prognostic clinic-pathologic features. The expression of MND1 increased significantly with advancing LUAD stage (OR = 1.68 for stage III vs. stage I, *p* = 0.046), suggesting that in patients with the elevated MND1 expression, LUAD was inclined to be diagnosed at a late stage. Age (continuous; OR = 0.37, *p* < 0.05) also was positively correlated with MND1 expression. No significant association of MND1 expression with distant metastasis (positive vs. negative; OR = 1.73, *p* = 0.209), lymph node metastasis (positive vs. negative; OR = 1.50, *p* = 0.098) and gender (male vs. female; OR = 1.34, *p* = 0.109; Table 2) was observed.

**Table 2:** Associations of MND1 expression with clinic-pathological features estimated by logistic regression analysis.

Clinical characteristics	Total (n)	OR for the elevated MND1 expression	*p*
Stage (III vs. I)	335	1.68 (1.01–2.83)	0.046
Distant metastasis (positive vs. negative)	348	1.73 (0.75–4.22)	0.209
Age (continuous)	458	0.37 (0.14–0.87)	0.028
Lymph node metastasis (positive vs. negative)	465	1.50 (0.93–2.43)	0.098
Gender (male vs. female)	477	1.34 (0.94–1.93)	0.109

Categorical dependent variable, greater or less than the median expression level.

The associations of MND1 expression level with survival rate and clinical stage are shown in Fig. 2C,D. LUAD samples were classified into high and low expression sets, and Kaplan–Meier curves based on the MND1 expression level were constructed (Fig. 2C). Log-rank test revealed that higher MND1 expression was significantly associated with poorer OS among LAUD patients compared with lower MND1 expression (*p* = 0.008). As shown in Fig. 2D, the expression level of MND1 in LUAD subjects with advanced stage disease (stage II–IV) was obviously higher than that in subjects with stage I disease (stage I vs stage II–IV, *p* = 0.009).

### MND1 expression, clinicopathological variables, and patient survival

The associations of OS with MND1 expression and clinic-pathological data were analyzed by Cox regression analyses (Table 3). HR results for OS were statistically significant based on the expression level of MND1 in all samples. As shown in Table 3, MND1 expression was significantly negatively correlated with OS (HR = 1.45, 95% CI 1.14–1.84, *p* = 0.002). Other clinic-pathologic variables including stage (HR = 1.65, 95% CI 1.40–1.95, *p* < 0.0001), T category (HR = 1.63, 95%CI 1.32–2.02, *p* < 0.0001), and N category (HR = 1.79, 95%CI 1.46–2.20, *p* < 0.0001) were significantly related to OS.

**Table 3:** Correlations of OS with clinic-pathologic characteristics in LUAD patients estimated using multivariate Cox regression analysis.

Clinicopathologic variable	OS	
	HR (95%CI)	*p* value
Age	1.00 (0.98–1.02)	0.843
Gender	1.04 (0.72–1.49)	0.852
Stage	1.65 (1.40–1.95)	0.000
T	1.63 (1.32–2.02)	0.000
M	1.76 (0.96–3.20)	0.066
N	1.79 (1.46–2.20)	0.000
MND1	1.45 (1.14–1.84)	0.002

*OS* overall survival, *HR* hazard ratio, *95%CI* 95% confidence interval.

**Figure 3:**
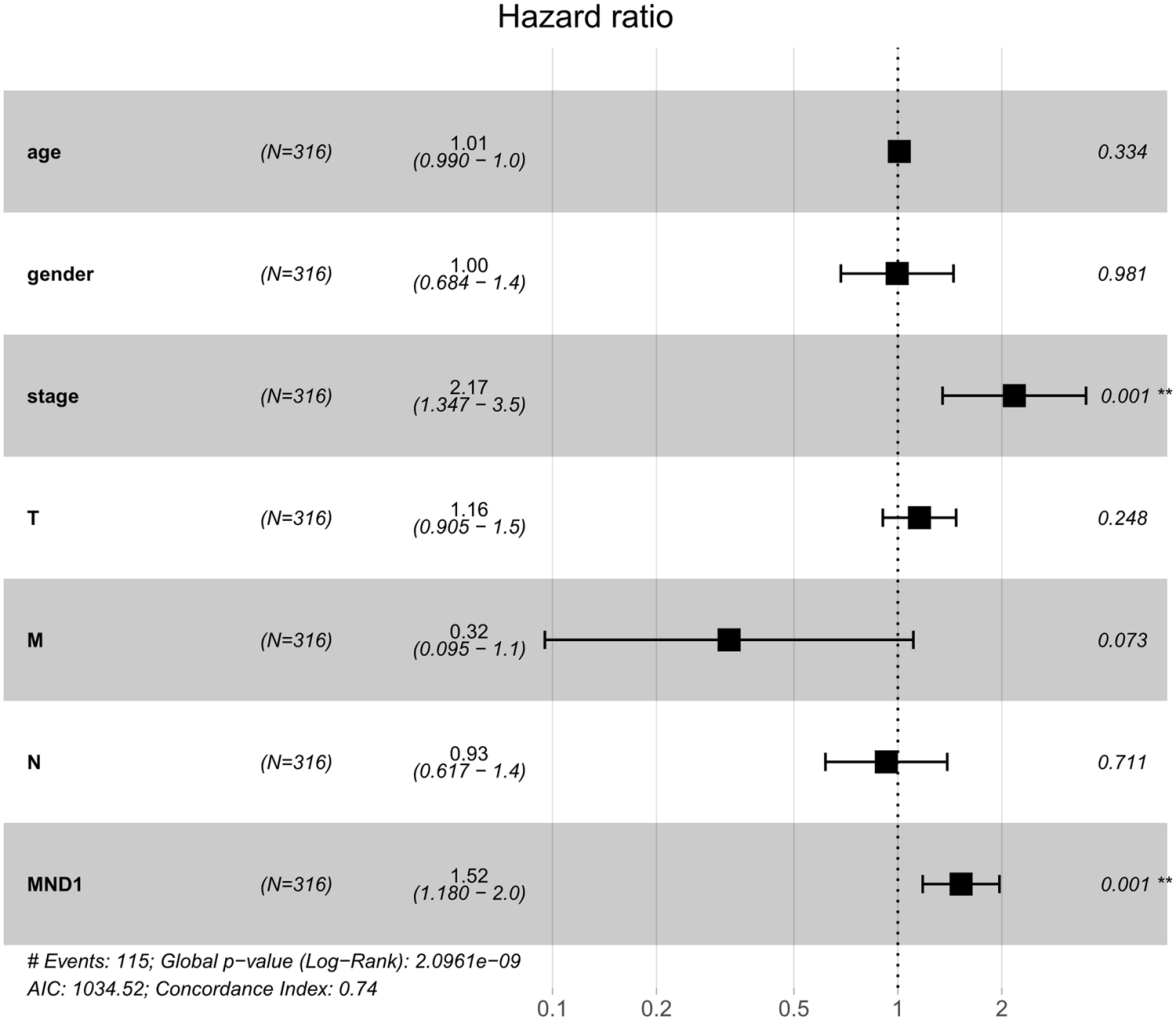
Forest plot from univariate Cox regression analysis of the associations of MND1 expression with clinic-pathological features.

In the univariate Cox regression model, categorical MND1 expression was significantly correlated with OS (HR = 1.52, 95%CI 1.18–1.97, *p* = 0.001, Table 4), as seen in the Forest plots obtained using the Survminer package in R (Fig. 3). However, clinicopathologic variables including age, gender, stage, and TMN classification were not significantly associated with OS. The results indicated that elevated expression of MND1 is independently correlated with OS in LUAD cases.

**Table 4:** Multivariate survival model after variable selection.

Clinicopathologic variable	OS	
	HR (95%CI)	*p* value
Age	1.01 (0.99–1.03)	0.334
Gender	1.00 (0.68–1.45)	0.981
Stage	2.17 (1.35–3.51)	0.001
T	1.16 (0.90–1.47)	0.248
M	0.32 (0.10–1.11)	0.073
N	0.93 (0.62–1.39)	0.711
MND1 expression	1.52 (1.18–1.97)	0.001

### GSEA identification of MND1-related signaling pathways

**Figure 4:**
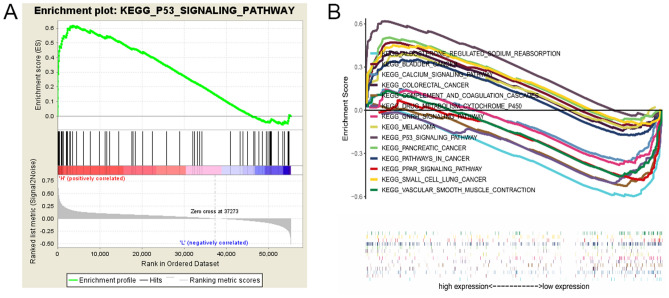
Enrichment plots from GSEA. (**A**) The p53 signaling pathway was differentially over-represented in LUAD cases with the elevated MND1 expression. (**B**) The p53 signaling pathway and pathways associated with pancreatic carcinoma, small cell lung carcinoma, bladder carcinoma, melanoma, and colorectal carcinoma were differentially over-represented in cases with the elevated MND1 expression, whereas the pathways for aldosterone-regulated sodium reabsorption, vascular smooth muscle contraction, PPAR signaling, complement and coagulation cascades, drug metabolism, cytochrome p450, calcium signaling and GNRH signaling were differentially over-represented in cases with low MND1 expression.

Signaling pathways that were differentially activated in LUAD patients were determined by GSEA via comparison of the high and low MND1 expression groups. Significant differences (false discovery rate [FDR] < 0.25, NOM-*p* < 0.05) were detected in enrichment of the Molecular Signatures Database (MSigDB) Collection. The most significantly enriched signaling pathways according to the NESs are shown in Fig. 4 and Tables 5 and 6. The most differentially over-represented signaling pathways in LUAD patients with the elevated MND1 expression included the p53 signaling pathway, pancreatic cancer, small cell lung cancer, bladder cancer, melanoma, and colorectal cancer (Table 5). Signaling pathways related to aldosterone-regulated sodium reabsorption, vascular smooth muscle contraction, peroxisome proliferator-activated receptor (PPAR) signaling pathway, complement and coagulation cascades, drug metabolism cytochrome p450, calcium signaling, and gonadotropin-releasing hormone (GNRH) signaling were differentially enriched in LUAD patients with low MND1 expression (Fig. 4, Table 6).

**Table 5:** Gene sets over-represented in the elevated MND1 expression phenotype.

Name	NES	NOM *p*-val	FDR q-val
KEGG_P53_SIGNALING_PATHWAY	2.304	0	0
KEGG_PANCREATIC_CANCER	1.881	0.006	0.016
KEGG_SMALL_CELL_LUNG_CANCER	1.674	0.019	0.072
KEGG_BLADDER_CANCER	1.622	0.023	0.092
KEGG_MELANOMA	1.485	0.042	0.15
KEGG_PATHWAYS_IN_CANCER	1.503	0.047	0.147
KEGG_COLORECTAL_CANCER	1.56	0.047	0.124

**Table 6:** Gene sets over-represented in low MND1 expression phenotype.

NAME	NES	NOM *p*-val	FDR q-val
KEGG_ALDOSTERONE_REGULATED_SODIUM_REABSORPTION	− 2.122	0	0.019
KEGG_VASCULAR_SMOOTH_MUSCLE_CONTRACTION	− 1.874	0	0.077
KEGG_PPAR_SIGNALING_PATHWAY	− 1.805	0.002	0.116
KEGG_COMPLEMENT_AND_COAGULATION_CASCADES	− 1.667	0.02	0.137
KEGG_DRUG_METABOLISM_CYTOCHROME_P450	− 1.643	0.021	0.14
KEGG_CALCIUM_SIGNALING_PATHWAY	− 1.533	0.029	0.159
KEGG_GNRH_SIGNALING_PATHWAY	− 1.529	0.027	0.156

## Discussion

LUAD is a highly complex and devastating disease for which the current therapeutic strategies are limited in number and largely ineffective. Consequently, considerable efforts have been made to develop novel and effective molecular-targeted therapeutic schemes. However, the molecular mechanisms underlying the progression and metastasis of LUAD as well as relevant biomarkers have yet to be determined. In the present study, a set of 495 DEGs was discovered from the GEO data and TCGA data, including 145 upregulated and 350 downregulated genes. Among them, the overlapping genes included MND1, and members of the p53 signaling pathway were significantly associated with LUAD, consistent with the results of Gao and Wang^16^ and Zhang et al.^17^, who reported several potential roles of MND1, cyclin family members and p53 signaling pathway proteins in LUAD^16,17^. Moreover, these significantly overlapping genes were key hub genes in the PPI network, with significant roles in the prognosis of LUAD, consistent with previous reports^16–18^. Among them, MND1 with 17 nodes (Fig. 1B) was predicted to connect with other important genes and pathways related to lung cancer, supporting the results of Dastsooz et al.^19^ and Zhang et al.^17^, who demonstrated the key role of MND1 expression and function in the occurrence and progress of carcinomas including LUAD.

DNA damage plays a causal role in numerous human pathologies including cancer, premature aging, and chronic inflammatory conditions. DNA repair pathway deficiencies have profound consequences on the signals of the immune system, eventually leading to malignant cancer^20,21^. In the present study, we discovered the association of hub gene MND1 with the occurrence and progression of LUAD. There have been few reports of MND1 expression in lung cancer^17^. MND1 is an intracellular protein that is expressed in the membrane of immune cells and cells of the thymus where it is essential in meiotic homologous chromosome pairing, synapsis and intragenic recombination during meiosis. The differential regulation of MND1 in LAUD patients and healthy controls is likely associated with the meiosis-specific HOP2-MND1 that form an extremely conserved and stable heterodimeric complex^22^ essential for homologous recombination in higher eukaryotes^23–25^. Homologous recombination repairs damaged chromosomes and mediates pairing of homologous chromosomes, playing a crucial role in maintaining telomere^26^ and genome stability^27^. Significantly, dysfunction in HR or its mediators and regulators can result in carcinoma-susceptible human diseases^28^. In the heterodimeric HOP2-MND1 complex, HOP2 acts as the major DNA-binding subunit, whereas MND1 is the important Rad51 interaction entity^22^ that modulates ATP and DNA binding by RAD51 to stabilize the RAD51 presynaptic filament and duplex DNA capture for enhancement of synaptic complex constitution. Chi et al.^22^ demonstrated stimulation of MND1 in both DMC1- and RAD51-mediated homologous strand assimilation, which is essential for the resolution of meiotic double-strand breaks. Furthermore, Bugreev et al. reported in vitro stimulation of HOP2-MND1 in the DNA strand exchange activities of RAD51 and DMC1^29^, leading to stabilization of the RAD51–single-stranded DNA nucleoprotein filament, the catalytic intermediate in recombination responses. The HOP2-MND1 complex that is predominantly expressed in human fibroblasts and cell lines^30^ acts in combination with RAD51 in recombination events to cause telomere lengthening. Alternations of HOP2 were detected in early onset familial breast and ovarian cancer subjects^31,32^, and a single amino acid deletion (Glu201 del) was associated with XX ovarian dysgenesis that is featured by streak ovaries^33^. Disruptions of Hop2 and MND1 gene expression are essential for DMC1 defects in homologous recombination^34^, whereas RAD51 loss is a functioning biomarker of the DNA damage in response to an unfavorable prognostic impact in non-small cell lung carcinoma cases undergoing curative surgical resection^35^. In the present study, the increased expression of MND1 in LUAD patients was associated with advanced clinical stage, short OS time, and poor prognosis, supporting the results of Dastsooz et al.^17^. Our study suggests that MND1 might be useful as a prognostic biomarker and treatment target for LUAD. Our findings need to be confirmed through further molecular validation studies as well as by clinical observations.

Weighted correlation network analysis (WGCNA) and gene set enrichment analysis (GSEA) have been widely used to identify classes of genes that are over-represented in a large set of genes and may have an association with disease phenotypes^36,37^. WGCNA is a co-expression network model for clustering analysis at the gene level, starting from the level of thousands of genes to determine the gene modules of clinical interest, and finally using the connectivity and gene importance within the modules to identify key genes in the disease pathway for further verification. Compared with GSEA, WGCNA provides more informative but nonsignificantly different results. However, the algorithm in WGCNA is more complicated and cumbersome for identifying modules corresponding to the biological approach. In the present study, we aimed to identify novel genes by performing clustering analysis of the biological functions of HUB genes rather than disease-related genes. Therefore, differential gene expression coupled with GSEA seems to more scientifically and accurately reflect the biological functions of genes. GSEA using TCGA data revealed that a set of important pathways including p53 signaling and pathways associated with malignancies such as pancreatic carcinoma, small cell lung carcinoma, bladder carcinoma, melanoma, and colorectal carcinoma were differentially over-represented in the elevated MND1 expression phenotype, whereas the pathways of aldosterone regulated sodium reabsorption, vascular smooth muscle contraction, PPAR signaling, complement and coagulation cascades, drug metabolism cytochrome P450, calcium signaling, and gonadotropin-releasing hormone (GnRH) signaling were differentially over-represented in the low MND1 expression phenotype. These data suggest that other genes identified by the GSEA as part of the MND1 protein network might play key roles in LUAD. Among the MND1-upregulated pathways, tumor suppressor p53 encoded by the homologous TP53 gene, has been previously proven to be involved in lung cancer, ranking first among all the genes detected in terms of its correlation with various types of human malignancies^38^. TP53 acts to slow down or monitor cell division^39^. Mutant p53 is a result of a TP53 gene alternation and acts as a tumor-promoting factor that functions essentially in the tumorigenesis of lung epithelial cells, resulting in cancer formation or cell transformation and elimination of normal TP53 gene functions^40^. Among the MND1-downregulated pathways, cytochrome p450 is a key enzyme in cancer formation and treatment^41^, serving important metabolic roles in a number of aspects of malignancy as a consequence of unusually broad substrate specificity. Cytochrome p450 is also a prominent player in the metabolism of anticancer therapy drugs to improve or diminish the drug efficacy, depending on whether the drug or its metabolites are effective. The cytochrome expression in lung carcinoma and surrounding tissues could be a crucial determiner of the efficacy of anticancer drugs^41,42^. In the present study, cytochrome p450 was inhibited by MND1, contributing to the longer OS of LUAD cases with low MND1 expression. Previous studies demonstrated correlations between TP53 mutation and poorer prognosis in non-small cell lung carcinoma^43^ and epidermal growth factor receptor (EGFR)-mutated LUAD^44–46^. However, the molecular functions of co-regulations between the targetable driver MND1 and tumor suppressor TP53 and other pathways such as cytochromes in the prognostic outcomes of LUAD patients have yet to be clarified. Multiplex genomic profiling datasets for LUAD patients are now available for machine-based-learning approaches^9,12^ and can be used to characterize both targetable driver alterations and tumor suppressor genes or pathways that are potentially significant for the design of therapeutic strategies and as predictive biomarkers for therapeutic efficacy in LUAD.

In conclusion, we discovered that MND1 expression is strongly negatively correlated with OS in LUAD patients. Notably, the effect of MND1 expression on the prognosis of LUAD patients is independent of clinicopathological features. Thus, MND1 can potentially serve as a prognostic biomarker for worse OS of LUAD patients and a target for the design of therapeutic schemes. Moreover, multiple pathways were upregulated or downregulated by MND1 expression during the occurrence and development of LUAD, among which the p53 signaling pathway might be the critical pathway through which MND1 modulated its effect on the OS of LUAD patients. However, our findings based on analyses of TCGA and GEO data in the present study need to be confirmed by analyses of biologically and functionally experimental data. The data regarding drug treatment for LUAD were also not available, limiting the analysis of clinical outcomes. Further in-depth studies are necessary to validate the results of the present study and reveal correlations between the targetable driver MND1 and the significant MND1-mediated pathways, which can then be applied to improve the therapeutic efficacy of treatments and prolong the OS of LUAD patients.

## Methods

### Data collection and process for differential expression analysis

Gene expression datasets GSE118370, GSE19188 and GSE40791 were downloaded from the GPL570 platform (https://www.ncbi.nlm.nih.gov/) and merged using Perlscript (ActivePerl-5.26.3.2603); these included data from 121 normal tissues and 118 LUAD tissues. The data were pre-processed for background adjustment and normalization by batch rectification using sva package (version 3.32.1) in R language (version 3.6.0;). The DEGs between LUAD and healthy tissues were analyzed with eBayes-test method of limma package (version 3.40.2) in R language^47^. P values were adjusted using the eBayes test, and an adjusted *p* value (adj.*p*) < 0.05 and |log FC|> 2 were used as the cutoff thresholds.

The clinicopathological features and transcriptomic profiles of LUAD cases were downloaded from TCGA (https://portal.gdc.cancer.gov/). Among 551 cases with transcriptome profiles, only LUAD tissues with full transcriptomic data and survival information were included, resulting in 486 clinical files for further analysis. Sixty-five samples (54 normal samples and 11 samples with incomplete clinicopathological features) were excluded. The clinicopathological features included pathological stage, age, gender, OS (survival days and survival state), TMN grouping, lymph node status, and distant metastasis status. All samples were tested by Illumina HiSeq 200 RNA Sequencing v.2 analysis. Fragments per kilobase of transcript per million mapped reads (FPKM) was applied as the unit of gene expression for categorization of LUAD cases into high and low expression sets. Variables in these two sets and the prevalence of categorical variables were compared using the Wilcox test. LUAD samples were divided into stage I and stage II–IV, according to the clinical phase at diagnosis.

### PPI network construction and hub gene analysis

The Search Tool for the Retrieval of Interacting Genes (STRING, https://string-db.org/) is an online database for retrieving interacting genes, including physical and functional correlations^48^. STRING^48^ was used to understand the protein–protein interaction by submitting the set of proteins and the respective pathway involved in LUAD was identified by using the Kyoto Encyclopedia of Genes and Genomes (KEGG) pathway database (https://www.kegg.jp/kegg/pathway.html)^49^. A STRING search was performed to evaluate the interactive associations among DEGs using a confidence score > 0.7 as the cut-off criterion, and significantly differentially expressed genes for the prognosis of LUAD were elected as hub genes (*p* < 0.05). Cytoscape software (version 3.5.1)^50^ was applied to construct PPI networks. The hub genes in the network were identified with cytoHubba (version 0.1)^51^ in Cytoscape to characterize crucial factors in LUAD.

### Gene set enrichment analysis (GSEA)

GSEA is a computational tool that allows the use of a priori gene sets to determine significantly over-represented or down-represented gene groups between two biological phenotypes^52^. The expression level of MND1 with 17 nodes in the PPI network was used as a phenotype label to measure the association between a set of genes and a phenotype in TCGA dataset. GSEA was carried out first to generate a sorted list of all genes based on their association with MND1 expression and then to assess whether survival differed significantly between the high- and low-MND1 expression groups using Java 8 (gsea-3.0.jar vision). Each gene set was repeatedly permutated 1000 times for each analysis. The nominal *p* value (NOM-*p*) and normalized enrichment score (NES) were applied to rank the pathways over-represented in each of phenotypes. GSEA enrichment plots were drawn using ggplot2 package in R.

### Statistical analysis

All data were statistically analyzed using R (v.3.6.0). The associations of clinic-pathological features, including age, gender, stage, and TMN grouping, with MND1 expression were estimated using the Wilcoxon signed-rank test and logistic regression. The associations of clinic-pathological characteristics with OS were identified using univariate Cox regression and multivariate Cox regression analyses. The multivariate Kaplan–Meier method in the R/Survminer package (version 0.4.4) was applied to generate the Kaplan–Meier survival plot. Multivariate Cox analysis was performed to determine the comparative effects of MND1 expression on survival among subgroups with different clinical parameters^53^: stage, lymph node status, distant metastasis status, age, and gender, using the median value of MND1 expression as the cut-off criteria. A hazard ratio (HR) based on the Cox PH model and the corresponding 95% confidence interval (95% CI) were estimated.

## Data Availability

The datasets generated and analyzed during the present study are available from the corresponding author upon reasonable request.
